# A review of advanced hydrogels for cartilage tissue engineering

**DOI:** 10.3389/fbioe.2024.1340893

**Published:** 2024-02-08

**Authors:** Mojtaba Ansari, Ahmad Darvishi, Alireza Sabzevari

**Affiliations:** Department of Biomedical Engineering, Meybod University, Meybod, Iran

**Keywords:** cartilage tissue engineering, injectable hydrogels, osteoarthritis (OA), drug delivery, mechanobiology

## Abstract

With the increase in weight and age of the population, the consumption of tobacco, inappropriate foods, and the reduction of sports activities in recent years, bone and joint diseases such as osteoarthritis (OA) have become more common in the world. From the past until now, various treatment strategies (e.g., microfracture treatment, Autologous Chondrocyte Implantation (ACI), and Mosaicplasty) have been investigated and studied for the prevention and treatment of this disease. However, these methods face problems such as being invasive, not fully repairing the tissue, and damaging the surrounding tissues. Tissue engineering, including cartilage tissue engineering, is one of the minimally invasive, innovative, and effective methods for the treatment and regeneration of damaged cartilage, which has attracted the attention of scientists in the fields of medicine and biomaterials engineering in the past several years. Hydrogels of different types with diverse properties have become desirable candidates for engineering and treating cartilage tissue. They can cover most of the shortcomings of other treatment methods and cause the least secondary damage to the patient. Besides using hydrogels as an ideal strategy, new drug delivery and treatment methods, such as targeted drug delivery and treatment through mechanical signaling, have been studied as interesting strategies. In this study, we review and discuss various types of hydrogels, biomaterials used for hydrogel manufacturing, cartilage-targeting drug delivery, and mechanosignaling as modern strategies for cartilage treatment.

## 1 Introduction

**FIGURE 1:**
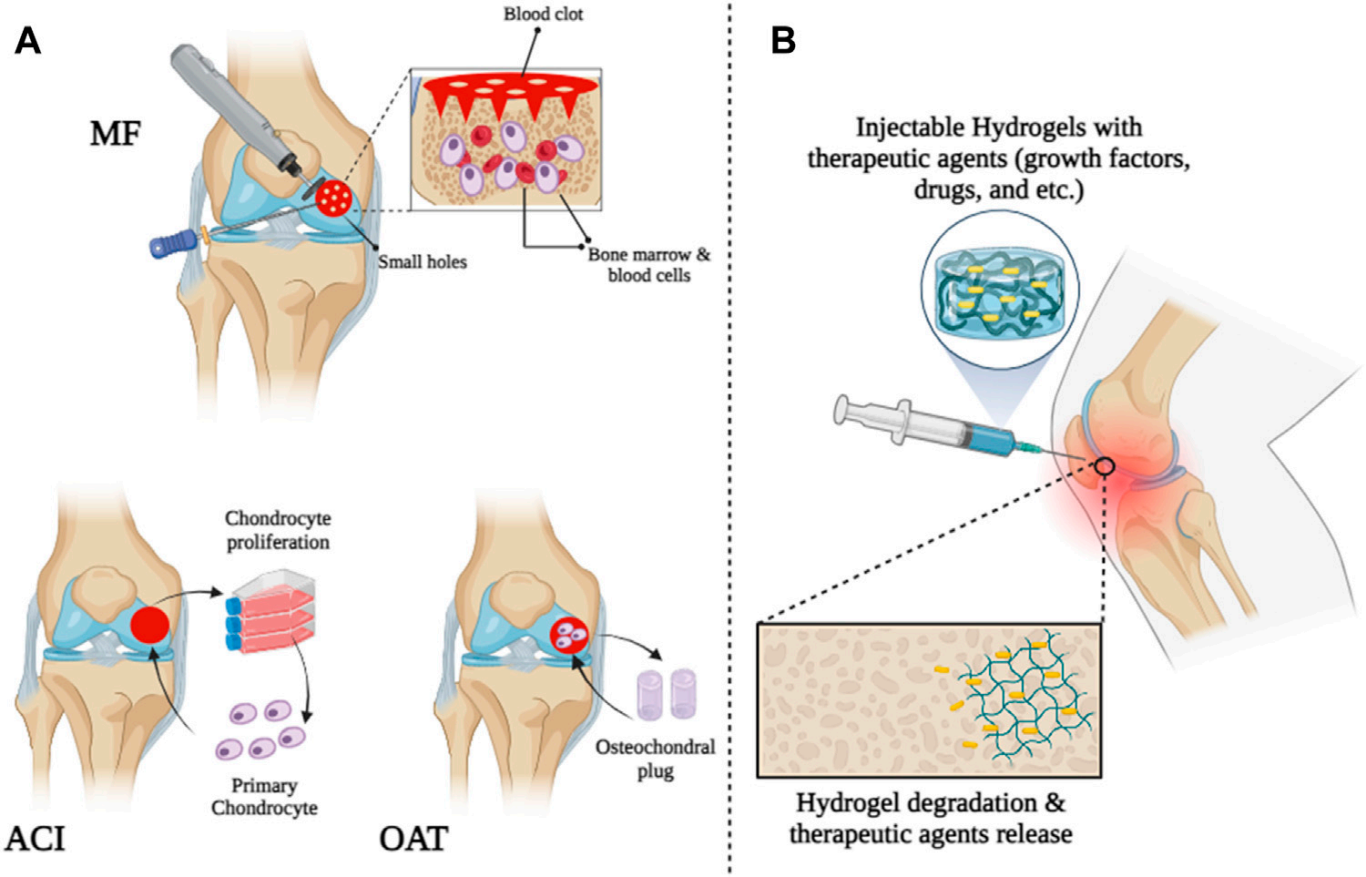
Schematic illustration of cartilage tissue treatment by **(A)** surgical strategies and **(B)** injectable hydrogels.

Damage to cartilage tissue can be caused by various conditions, including sports injuries, arthritis, and trauma (Walker and Madihally, 2015; Söntjens et al., 2006; Ren K. et al., 2015; Cancedda et al., 2003). It has been reported that 15% of people over 60 years of age and 60% of patients who undergo knee arthroscopy suffer from cartilage damage (Hjelle et al., 2002; Vilela et al., 2015). Cartilage tissue has no nerves or blood vessels and survives by receiving oxygen and nutrients from synovial fluid. Because of this, recovery of cartilage tissue is difficult (if damaged) (Giannoudis et al., 2005; Sen and Miclau, 2007; Kim T. G. et al., 2012; Cully, 2013; Flierl et al., 2013; Marenzana and Arnett, 2013). Medical repair of damaged cartilage tissue is still necessary. Therefore, the development of a method that can permanently and completely treat cartilage tissue in patients with cartilage damage is very clinically important. Cartilage defects are treated with some surgical methods such as microfracture surgery, Autologous Chondrocyte Implantation (ACI), and Mosaicplasty. A small hole is created in the subchondral bone when repairing a microfracture beneath a cartilage defect. When a hole occurs, bone marrow cells and blood cells leave the hole and form a blood clot on the surface, allowing the cells that form cartilage to escape and repair the damage (Yan et al., 2020). This method is inexpensive and simple, but its long-term effectiveness has not been reported. Additionally, the possibility of side effects such as osteophyte formation, cysts, and bone overgrowth after microfracture surgery limits the use of this technique (Sun et al., 2020; Kim M. S. et al., 2021). Another surgical method for repairing full-thickness defects of articular cartilage, which has also received the approval of the US Food and Drug Administration (FDA), is called Autologous chondrocyte implantation (ACI). In this method, the surgeon first removes parts of the healthy articular cartilage that do not bear much load. Then, chondrocytes are implanted on these sections for 4 weeks in a laboratory environment. Finally, new cartilage sections are implanted in the damaged areas and chondrocytes regenerate new tissue (Marlovits et al., 2006; Guillen-Garcia et al., 2023). Osteochondral autograft transfer (OAT) or mosaicplasty, is another new and proven surgical method for treating cartilage defects in patients. In this method, cylindrical pieces from the parts that bear less load are removed from healthy cartilage by the surgeon and placed in the damaged area. Although this method has been proven to treat articular cartilage defects, this method cannot repair large defects (Figure 1A) (Kowalczuk et al., 2018).

The advent of tissue engineering in 1990 gave scientists hope for repairing and regenerating damaged cartilage tissue (Grottkau and Lin, 2013; Sahni et al., 2015; Bush et al., 2016; Wang et al., 2016). Engineered tissues are composed of a scaffold, essential growth factors, and cells (Balakrishnan and Banerjee, 2011; Huang et al., 2014). In general, engineered cartilage tissue scaffolds should possess properties such as porosity, non-toxicity, favorable biocompatibility, cell differentiation, and new tissue regeneration. Additionally, these scaffolds must be able to degrade after tissue regeneration, have an appropriate release rate of nutrients and metabolites, have stable mechanical properties, and be able to attach to the surrounding tissue and fill in the damaged area (Hollister, 2005; Seliktar, 2012; Huang et al., 2014; Zhang et al., 2014; Ren K. et al., 2015).

Since the 1990s, various biomaterials have been studied and tested for use in cartilage tissue engineering (Drury and Mooney, 2003; Slaughter et al., 2009; Zhang and Webster, 2009; Zhang et al., 2012; Deng et al., 2013; Choi et al., 2014; Fan et al., 2015; Fan and Wang, 2015; Lu et al., 2015; Shin et al., 2016). Among all biomaterials, hydrogels have received extensive attention as scaffolds for cartilage tissue engineering due to their porous scaffolds and structural similarities to extracellular matrix (ECM) (Van Vlierberghe et al., 2011). Hydrogels are hydrophilic three-dimensional structures composed of homopolymers and copolymers that can absorb water and swell in aqueous solutions, creating an appropriate microenvironment similar to ECM (Kabiri et al., 2016; Sabzevari et al., 2016; Sabzevari and Kabiri, 2016). Therefore, it promotes the attachment, migration, differentiation, and proliferation of osteoblasts and chondrocytes and effectively delivers growth factors and nutrients (Jin et al., 2009; Van Vlierberghe et al., 2011; Yazdimamaghani et al., 2014; Daly et al., 2020). Traditionally, bulk hydrogels are crosslinked with external dimensions of millimeters or more and cell sizes of nanometers to allow for molecular diffusion. However, bulk hydrogels are not always suitable for their intended use, especially when smaller sizes are required or injections are required (Sivashanmugam et al., 2015). Recently, researchers have studied and explored injectable hydrogels in cartilage tissue engineering due to their ability to adapt to irregular tissue defects and give good and desired shapes, replacing risky and invasive surgeries with less invasive methods (Figure 1B) (Wei et al., 2008; Gong et al., 2009; Tan et al., 2011; Bidarra et al., 2014; Ren K. et al., 2015; Shen et al., 2015). Injectable hydrogels are synthesized using a variety of natural and artificial biomaterials. These biomaterials include chitosan (Tan et al., 2011), alginate (Hong et al., 2008), collagen or gelatin (Dorsey et al., 2015; Sim et al., 2015), heparin (Alexander et al., 2013), hyaluronic acid (Wang et al., 2010), poly (ethylene glycol) (PEG) (Ossipov et al., 2008), chondroitin sulfate (Lin et al., 2015), and polyvinyl alcohol (PVA) (Jin et al., 2010a). Depending on the production method, injectable hydrogels can be classified into several categories: Photo-crosslinked hydrogels (Tan and Marra, 2010), enzyme-linked hydrogels (Li et al., 2012), Michael addition-mediated hydrogels (Shinde et al., 2013), Schiff-based crosslinked hydrogels (Chiu et al., 2009), click chemistry-mediated hydrogels, Gong et al. (2009), Park et al. (2014) pH-sensitive hydrogels (Choi et al., 2011), ion-sensitive hydrogels, Yeon et al. (2013) temperature-sensitive hydrogels (Sideris et al., 2016; Mealy et al., 2018), and hydrogel microparticles (HMPs) or HMP microgels (Sivashanmugam et al., 2015) with the following unique properties; Their small size (can be administered through catheters and small needles), significant porosity, and modular nature make them suitable for biomedical applications (Table 1) (Sivashanmugam et al., 2015).

**TABLE 1:** Studies on injectable hydrogels in cartilage tissue engineering.

Materials	Major outcomes	Ref.
collagen types I and II	chondrocytes implanted in hydrogels secrete cartilage-specific ECM.	Yuan et al. (2016)
collagen type II	hyaline cartilage showed good regeneration after 8 weeks, and there was a significant difference in cartilage regeneration between the control group and the transplant group after 24 weeks	Funayama et al. (2008)
collagen type II and hyaluronic acid	induce proliferation and survival of chondrocytes	Kontturi et al. (2014)
aminogelatin, four-strand PEG acrylate, and oxidized dextran	proliferation and expansion of chondrocyte cells after embedding them in the produced injectable hydrogel	Geng et al. (2012)
methacryloyl gelatin modified with poly-L-lysine and phenylboronic acid (Gel-EPL/B)	increased the differentiation of stem cells into chondrocytes, promoted the deposition of the extracellular matrix of chondrocytes, and created a 3D microenvironment for cartilage repair	Wang et al. (2021)
calcium-phosphate-alginate	better cell viability, bone differentiation, and mechanical properties	Zhao et al. (2010)
alginate/hyaluronic acid	Biodegradability, cartilage regeneration (6 weeks after injection in mice	Park and Lee (2014)
oxidized periodate alginate, gelatin and borax	excellent cell viability, cell migration and proliferation, low inflammatory response, and good integration with cartilage tissue	Balakrishnan et al. (2014)
alginate, O-carboxymethyl chitosan, and fibrin nanoparticles	Suitable mechanical properties, swelling rate, biodegradability, and biocompatibility	Jaikumar et al. (2015)
glycerol phosphate, chitosan, and hydroxyethylcellulose	Favorable cell viability, proliferation, and differentiation	Naderi- et al. (2014)
starch, N-succinyl, chitosan, and dialdehyde	limited water absorption, more robust structure, lower weight loss, and shorter gelation time	Kamoun (2016)
heparin-tyramine and dextran-tyramine	proliferation of chondrocytes, and increased production of collagen and chondroitin sulfate	Jin et al. (2011)
Heparin, gelatin (L-lactide-co-ε-caprolactone)	increased glycosaminoglycan production, repair of damaged cartilage tissue, and formation of new tissue that could integrate with normal cartilage tissue	Kim et al. (2012b)
chondroitin sulfate/poly (N-isopropylacrylamide)	no cytotoxicity (tested on 293 human fetal kidney cells), excellent adhesion to surrounding tissue, increased tensile strength (from 0.4 to 1 kPa), and improved mechanical properties	Wiltsey et al. (2013)
chondroitin sulfate/pullulan	increased cell proliferation, high cytocompatibility, and cartilaginous ECM deposition	Chen et al. (2016)
hyaluronic acid/PEG	high mechanical properties (breaking strength = 109.4 kPa, storage modulus = 27 kPa and compressive strain 81.9%), cell viability and proliferation	Yu et al. (2014a)
hyaluronic acid/chitosan	excellent biocompatibility, high cell proliferation and increased ECM deposition in cartilage	Barbucci et al. (2002)
hyaluronic acid derivatives (particularly ethylene diamino and amino/octadecyl hyaluronic acid), and divinyl sulfone with functionalized inulin	improved the mechanical properties (elastic modulus 14.8 ± 0.6 kPa), reduced hydrogels susceptibility to hydrolysis by hyaluronidase	Palumbo et al. (2015)
heparin-conjugated fibrin	appropriate biodegradability (within 4 weeks) and sustained release of BMP-4 and TGF-β1. Increased subchondral bone and hyaline cartilage regeneration compared to the control sample (over 12 weeks)	Sarsenova et al. (2022)
fibrin/agarose	The prepared artificial cartilage showed high cell compatibility and mechanical stability	Bonhome-Espinosa et al. (2020)
elastin-like recombinamer (ELR)	ELR hydrogels only regenerated hyaline cartilage. Hydrogels embedded with rMSCs also result in proper bone regeneration	Cipriani et al. (2019)
elastin-like recombinamer (ELR)	ELR-hMSCs hydrogel caused the complete formation of hyaline cartilage and subchondral bone	Pescador et al. (2017)
poly (L-glutamic acid)	favorable mechanical properties, rapid gelation, well injectability, and high cell viability and proliferation	Yan et al. (2016)
PEG	adequate cartilage regeneration	Skaalure et al. (2015)
hyaluronic acid and PEG	short gelation times, favorable mechanical properties, and high cell viability and proliferation	Yu et al. (2014b)

In this study, we first investigated injectable hydrogels for cartilage engineering. We will then look at some biomaterials and different methods for making injectable hydrogels. Finally, we will review other existing approaches used in cartilage tissue engineering, such as targeted drug delivery.

## 2 Injectable hydrogels for cartilage tissue engineering

### 2.1 Biomaterials used to make injectable hydrogels

Among the available biomaterials, two main types (natural and synthetic) are used to fabricate hydrogels and scaffolds in cartilage tissue engineering (Ansari and Eshghanmalek, 2019). In this section, we will look at some of these biomaterials (both synthetic and natural).

#### 2.1.1 Injectable hydrogels based on natural polymers

Natural biomaterials recently studied and used for the production of injectable hydrogels include collagen/gelatin, alginate, chitosan, heparin, chondroitin sulfate, and hyaluronic acid. Of course, other natural biomaterials, such as fibrin or elastin, have been reported to be used to create injectable hydrogels, but these are not discussed in this article.

##### 2.1.1.1 Collagen/gelatin-based injectable hydrogel

There are a total of 28 types of collagens in the mammalian body (e.g., collagen types I, II, III, and IV), found in large quantities in tissues such as cartilage, bones, ligaments, skin, and connective tissue (Lee et al., 2001; Ackermann and Steinmeyer, 2005; Rubert Pérez et al., 2011; Parmar et al., 2015; Bielajew et al., 2020). Recently, due to the weak antigenicity of collagen, collagen-derived natural biomaterials have been explored and used for various applications in tissue engineering, especially cartilage tissue engineering, such as the synthesis and production of collagen-based hydrogels (Yuan et al., 2016). For example, in one study, collagen types I and II were used to create injectable hydrogels in cartilage tissue engineering. This study by Yuan et al. showed that the compressive modulus of hydrogels can be tuned by changing the collagen type I content. Additionally, they showed that chondrocytes implanted in hydrogels secrete cartilage-specific ECM and maintain normal morphology (Yuan et al., 2016). In another study, Funayama et al., 2008 created an injectable type II collagen hydrogel and injected it into damaged rabbit cartilage. As a result, hyaline cartilage showed good regeneration after 8 weeks, and there was a significant difference in cartilage regeneration between the control group and the transplant group after 24 weeks. Konturi et al. combined type II collagen and hyaluronic acid to create an injectable hydrogel for damaged cartilage regeneration. Cell morphology, proliferation and viability, gene expression, and glycosaminoglycan production were examined. As a result of the study, this hydrogel was shown to induce proliferation and survival of chondrocytes, and as a result, it could be a suitable injectable hydrogel for cartilage tissue engineering (Kontturi et al., 2014).

Collagen is broken down to obtain gelatin. It is a natural protein with excellent biodegradability and biocompatibility in the body’s physiological space (Santoro et al., 2014; Song et al., 2015). In the past few years, the use of gelatin for the synthesis and production of injectable hydrogels in cartilage tissue engineering has been explored. For example, Geng et al., 2012 fabricated injectable gelatin-based hydrogels from aminogelatin, four-strand PEG acrylate, and oxidized dextran through a two-step process. Cell culture studies showed the proliferation and expansion of chondrocyte cells after embedding them in the produced injectable hydrogel. These results confirmed the biocompatibility and biodegradability of the hydrogel. In another study, Wang et al. synthesized an injectable hydrogel based on methacryloyl gelatin modified with poly-L-lysine and phenylboronic acid (Gel-EPL/B) for cartilage defect repair. *In vitro* and *in vivo* evaluation results showed that Gel-EPL/B hydrogel shows better biocompatibility compared to the control group (GelMA). In addition, Gel-EPL/B hydrogel increased the differentiation of stem cells into chondrocytes, promoted the deposition of the extracellular matrix of chondrocytes, and created a 3D microenvironment for cartilage repair (Wang et al., 2021).

##### 2.1.1.2 Alginate-based injectable hydrogel

Alginate is a polysaccharide extracted from brown algae (Phaeophyceae) and consists of mannuronic acid and guluronic acid (Venkatesan et al., 2015; Zhang et al., 2015). Alginate is one of the most common biomaterials used for fabrication of injectable hydrogels in cartilage tissue engineering due to its non-toxicity, non-immunogenicity, and favorable morphogenic ability (Park et al., 2009; Follin et al., 2015; Venkatesan et al., 2015; Ruvinov and Cohen, 2016). However, alginate-based injectable hydrogels have a serious problem in that they cannot maintain the structure of the regenerated tissue (Kretlow et al., 2009). Therefore, to improve the mechanical properties of alginate-based hydrogels, they are modified or combined with various biomaterials. For example, an injectable cement-phosphate-alginate hydrogel was prepared during the study. Results showed that injectable calcium phosphate alginate hydrogel cementitious hydrogels provided significantly better cell viability, bone differentiation, and mechanical properties than previously injected samples (Zhao et al., 2010). Additionally, because alginate lacks cell adhesive properties, it is commonly combined with other polymers. Accordingly, Park and Lee prepared an injectable alginate/hyaluronic acid hydrogel and investigated its properties. Results showed a biodegradable hydrogel with cartilage regeneration capabilities (6 weeks after injection in mice) (Park and Lee, 2014). In another study, Balakrishnan et al., 2014 prepared a hydrogel with a fast gelation rate using oxidized periodate alginate and gelatin in the presence of borax. The evaluation showed that the resulting injectable hydrogel offers excellent cell viability, cell migration and proliferation, low inflammatory response, and good integration with cartilage tissue. In another study, a combination of alginate, O-carboxymethyl chitosan, and fibrin nanoparticles was used to prepare an injectable biodegradable hydrogel. As a result of studying the mechanical properties, swelling rate, biodegradability, and biocompatibility, it was found that the alginate/O-carboxymethyl chitosan-based injectable hydrogel could be used as a suitable composition for cartilage tissue engineering (Jaikumar et al., 2015).

##### 2.1.1.3 Chitosan-based injectable hydrogel

Natural chitin is composed of N-acetylglucosamine and glucosamine. Chitosan is a polysaccharide with a linear structure obtained from natural chitin (Di Martino et al., 2005; Tan et al., 2009; Yang et al., 2009; Hu et al., 2015a; Siahkamari et al., 2017). In recent studies, the production of injectable hydrogels in cartilage tissue engineering has attracted attention due to the structural similarity of chitosan with glycosaminoglycans of cartilage tissue (Di Martino et al., 2005; Naderi-et al., 2014). For example, in one study, glycerol phosphate, chitosan, and hydroxyethylcellulose as a cross-linker were used to prepare an injectable hydrogel for cartilage repair. Assessment showed cell viability, proliferation, and differentiation. Therefore, this hydrogel can be used in the field of cartilage tissue engineering (Naderi-et al., 2014). Chitosan has also been studied and used in the field of producing injectable, stimuli-responsive hydrogels. For example, one study combined glycerophosphate/chitosan with various concentrations of starch to prepare thermosensitive injectable hydrogels for cell delivery (Sá-Lima et al., 2010). Chitosan is soluble in acetic acid solution. At the same time, one of the problems with chitosan-based hydrogels is that chitosan is insoluble in water (Yang et al., 2008). To address this problem, Kamoun created an injectable hybrid hydrogel of starch, N-succinyl, chitosan, and dialdehyde with biodegradable and non-toxic properties (Kamoun, 2016). The evaluation showed that this hydrogel offers limited water absorption, more robust structure, lower weight loss, and shorter gelation time. The researchers found that the aforementioned properties strongly depend on the ratio of N-succinyl and dialdehyde polymers, where with the increase in the ratio of N-succinyl (SCS) in the hybrid hydrogel compared to dialdehyde (DAS), the gelation time became faster and shorter (SCS: DAS = 9:1; 10 min, and SCS: DAS = 1:5; 80 min). These results can be attributed to the higher molecular weight of SCS compared to DAS. Likewise, SEM evaluation results showed that with increasing SCS ratio, the hydrogel structure is stronger and the size of fine pores decreases, but with increasing DAS, a loose and honeycomb structure with more hydrophilic capacity is prepared. In addition, it was shown that the high content of SCS compared to DAS caused the formation of a hydrogel with a more compact structure and stronger cross-linking, which reduced the rate of hydrolysis and weight loss of the hydrogel in PBS. The increased rate of weight loss in the hydrogel with high DAS was attributed to the enzymatic degradation of starch by the α-amylase enzyme. Therefore, it can be used as an injectable chitosan-based hydrogel (with limited water absorption) for cartilage tissue engineering applications (Kamoun, 2016).

##### 2.1.1.4 Heparin-based injectable hydrogel

Heparin is a negatively charged, highly sulfated linear polysaccharide composed of repeating disaccharide units of 1,4-uronic acid and glucosamine and has anticoagulant properties (Casu, 1985; Sundaram et al., 2003; Tae et al., 2007; Liang and Kiick, 2014). Due to the negative charge of the functional groups, heparin can play a role in cell proliferation and differentiation as well as the initiation of signaling pathways and is associated with growth factors and ECM proteins (Go et al., 2008; Guillame-et al., 2010; Hudalla and Murphy, 2011; Mammadov et al., 2012; Wang et al., 2013; Yang et al., 2014). As a result, heparin is used to prepare injectable hydrogels for various applications such as cartilage tissue engineering, growth factor and protein transport (Nakamura et al., 2006; Go et al., 2008). For example, one study used horseradish peroxidase (HRP) to prepare injectable heparin-tyramine and dextran-tyramine (Dex-TA) hydrogels. Evaluation of the swelling, mechanical properties, viability, and proliferation of chondrocytes along with increased production of collagen and chondroitin sulfate showed that these hydrogels can be used for cartilage tissue engineering (Jin et al., 2011). Additionally, a strategy to increase the therapeutic effect is to combine heparin-based hydrogels with other scaffolds (hydrogels/scaffolds). In one study, a hydrogel/scaffold composite was prepared using heparin-based hydrogel and a porous scaffold containing gelatin (L-lactide-co-ε-caprolactone). *In vivo* evaluation showed increased glycosaminoglycan production, repair of damaged cartilage tissue, and formation of new tissue that could integrate with normal cartilage tissue (Kim M. et al., 2012). These results show that hydrogel/scaffold composites can be implemented as promising systems in cartilage tissue engineering.

##### 2.1.1.5 Chondroitin sulfate-based injectable hydrogel

It is an anionic polysaccharide of linear structure, formed from sequential sulfated disaccharide units with 1–3 linkages of D-glucuronic acid and N-acetylgalactosamine, and is present in bone, cartilage and connective tissue (Knutson et al., 1996; Wang et al., 2007). Chondroitin sulfate plays an effective role in many biological tasks, such as cell identification, regulation of chondrocyte phenotype, intracellular signaling, communication between cell surface glycoproteins and ECM components, and cartilage tissue engineering (Strehin et al., 2010; Zhang L. et al., 2011; Jo et al., 2012; Liao et al., 2015). For example, the study used a chondroitin sulfate/poly (N-isopropylacrylamide) combination to create an injectable hydrogel. Evaluation of the hydrogel showed no cytotoxicity (tested on 293 human fetal kidney cells), excellent adhesion to surrounding tissue, increased tensile strength (from 0.4 to 1 kPa), and improved mechanical properties (Wiltsey et al., 2013). Another study prepared a composite injectable hydrogel containing a chondroitin sulfate/pullulan combination to repair damaged cartilage tissue. Evaluation showed increased cell proliferation, high cytocompatibility, and cartilaginous ECM deposition. Therefore, it can be used for cartilage tissue engineering (Chen et al., 2016).

##### 2.1.1.6 Hyaluronic acid-based injectable hydrogel

It is a linear polysaccharide in the ECM of adult cartilage composed of the disaccharide units N-acetylglucosamine and glucuronic acid (Camenisch and McDonald, 2000; Muzzarelli et al., 2012). Hyaluronic acid binds to cartilage cells (chondrocytes) through surface receptors such as receptor for hyaluronan-mediated motility (RHAMM) and CD44 (Evanko et al., 2007; Kim et al., 2011). Additionally, hyaluronic acid plays a role in mesenchymal cell density, chondrogenic differentiation, cartilage matrix deposition, and finally chondrogenesis (Knudson, 2003; Astachov et al., 2011). Therefore, hyaluronic acid can be used as a suitable and ideal biomaterial in cartilage tissue engineering. For example, in one study, we prepared an injectable hyaluronic acid/PEG hydrogel for use in cartilage tissue engineering. Evaluation results showed high mechanical properties (breaking strength = 109.4 kPa, storage modulus = 27 kPa and compressive strain 81.9%), cell viability and proliferation (Yu et al., 2014a). In another study, an injectable hyaluronic acid/chitosan hydrogel was prepared using methacrylate glycol chitosan and hyaluronic acid and considering the ionic complexation of chitosan and structural similarity to glycosaminoglycans. Results showed excellent biocompatibility, high cell proliferation and increased ECM deposition in cartilage (Barbucci et al., 2002). In general, hyaluronic acid is modified or combined with other biomaterials to solve problems such as rapid decomposition, hydrolysis reaction, and weak mechanical properties of hyaluronic acid (Palumbo et al., 2015; Antons et al., 2018). For example, Palumbo et al., 2015 created *in situ* hydrogels using hyaluronic acid derivatives (particularly ethylene diamino and amino/octadecyl hyaluronic acid) and added divinyl sulfone with functionalized inulin. The results showed that the presence of C18 pendant chains improved the mechanical properties of hyaluronic acid hydrogels (elastic modulus 14.8 ± 0.6 kPa) and also reduced their susceptibility to hydrolysis by hyaluronidase. Additionally, these hydrogels showed high cell viability and proliferation. All these results show that hyaluronic acid can be used as a suitable natural biomaterial with potential applications in cartilage tissue engineering.

##### 2.1.1.7 Fibrin-based injectable hydrogel

Fibrin is a natural biopolymer derived from fibrinogen in the presence of thrombin. Fibrinogen is present in the blood and plays an important role in homeostasis, inflammation, angiogenesis, differentiation, proliferation, migration, and cell adhesion. During blood clotting, thrombin cleaves fibrinopeptides A and B (FpA and FpB) from the N-terminal site of fibrinogen chains and forms fibrin polymer (Harris and Marles-Wright, 2021; Rojas-Murillo et al., 2022). Fibrin-based scaffolds with appropriate elasticity, balanced mechanical strength, mesh-like structure, biodegradability, and excellent biocompatibility have shown great potential for use in cartilage tissue engineering applications. Generally, these structures are available in three forms; Hydrogels, adhesives, and microbeads. Previously, fibrin adhesives were used during surgery in articular cartilage. With the advancement of technology, the use of fibrin-based hydrogels to repair cartilage defects has become more popular. These hydrogels are composed of calcium salt, thrombin, and fibrinogen (Noori et al., 2017; Rojas-Murillo et al., 2022). The effective application of fibrin-based hydrogels both *in vitro* and *in vivo* for cartilage tissue engineering has been proved. For example, in one study, a heparin-conjugated fibrin (HCF)-based hydrogel was prepared to repair cartilage defects in a rabbit model. Synovium-derived mesenchymal stem cells (SDMSCs), bone morphogenic protein 4 (BMP-4), and transforming growth factor beta 1 (TGF-beta 1) were encapsulated in hydrogel. *In vitro* studies showed that the HCF hydrogel provides appropriate biodegradability (within 4 weeks) and sustained release of BMP-4 and TGF-β1. In addition, pathobiological and *in vivo* studies showed that implantation of HCF hydrogel encapsulated with SDMSCs, BMP-4, and TGF-β1 increased subchondral bone and hyaline cartilage regeneration compared to the control sample (over 12 weeks) (Sarsenova et al., 2022). Campos et al. prepared and evaluated a 3D magnetic fibrin/agarose-based hydrogel by encapsulating human hyaline chondrocytes and magnetic nanoparticles as artificial cartilage tissue. The rheological results showed that the presence of magnetic nanoparticles increases the storage modulus and loss modulus of hydrogels at different times. Moreover, immunohistochemical evaluation did not rule out the expression of type II collagen in human hyaline chondrocytes. In addition, the prepared artificial cartilage showed high cell compatibility and mechanical stability. According to the research results, the prepared hydrogel can be considered and used as an engineered hyaline cartilage tissue (Bonhome-Espinosa et al., 2020). Finally, the use of fibrin as a natural biomaterial is considered a promising approach in cartilage defect repair.

##### 2.1.1.8 Elastin-based injectable hydrogel

Elastin is a protein biopolymer that is mostly found in soft tissues such as skin, lungs, and blood vessels. It is a water-insoluble (hydrophobic) biopolymer. Likewise, it can induce cell-cell interaction and increase tissue elasticity. Therefore, elastin-based biomaterials have been widely evaluated and used for tissue engineering applications, especially cartilage tissue (Audelo et al., 2020; Varanko et al., 2020). For example, in a study, injectable hydrogels based on elastin-like recombinamer (ELR) were prepared and rabbit mesenchymal stromal cells (rMSCs) were embedded in them. The prepared hydrogels were injected in 10 New Zealand rabbits with subchondral defects for 4 months. The results showed that ELR hydrogels only regenerated hyaline cartilage, which was reported due to the presence of elastin. Hydrogels embedded with rMSCs also result in proper bone regeneration (Cipriani et al., 2019). In a similar study, an ELR-based injectable hydrogel loaded with human mesenchymal stem cells (hMSCs) was developed to promote osteochondral regeneration. The results were evaluated after injection of hydrogels for 3 months in rabbit thighs. It was shown that the ELR-hMSCs hydrogel caused the complete formation of hyaline cartilage and subchondral bone (Pescador et al., 2017). Therefore, it can be said that the use of elastin-based hydrogels is a new approach to induce the transformation of stem cells into chondrocytes and to repair subchondral defects.

#### 2.1.2 Injectable hydrogels based on synthetic polymers

Synthetic polymers have been used to study cell-matrix interactions and cartilage tissue engineering due to their improved reproducibility, controllability, and degradability. Several synthetic biomaterials currently used for cartilage tissue engineering include polyvinyl alcohol (PVA) (Bonakdar et al., 2010), PEG (Yang et al., 2015; Fan et al., 2016), polypropylene fumarate (PPF) (Kallukalam et al., 2008), poly (L-glutamic acid) (Yan et al., 2014; Yang et al., 2016), α,β-poly-(N-hydroxyethyl)-DL-aspartamide (Sun et al., 2009), methoxypolyethylene glycol-poly (ε-caprolactone) (Kwon et al., 2013a), and PEG-poly (N-isopropylacrylamide) (PNIPAAm) (Alexander et al., 2014). Among these polymers, PVA has been recognized as a promising candidate for cartilage repair due to its unique properties such as non-toxicity, good biocompatibility, and water solubility (Semsarzadeh and Sabzevari, 2018; Semsarzadeh and Sabzevari, 2020; Chen et al., 2021; Sabzevari et al., 2023). Well-defined PVA can be easily synthesized using cobalt-mediated radical polymerization of vinyl acetate and then hydrolysis of polyvinyl acetate (Semsarzadeh et al., 2018; Sabzevari et al., 2022a; Sabzevari et al., 2022b; Sabzevari et al., 2023). Like PVA, polyethylene glycol (PEG) is a hydrophilic polymer that is highly soluble in water. Additionally, these polymers are used in applications such as fabrication of injectable hydrogels in tissue engineering due to their unique properties such as suitable biocompatibility, non-toxicity, anti-protein absorption, and non-immunological effects (Liu et al., 2019; Gan et al., 2021). Additionally, some PEG derivatives are sensitive to pH stimulation and can therefore be used to create smart pH-responsive hydrogels (Kono, 2014; Gong et al., 2018). Injectable PEG-based hydrogels can be prepared using methods such as functional group reaction, photopolymerization, and free radical polymerization (Day et al., 2018; Wang et al., 2018; Jiang et al., 2020).

One study used poly (L-glutamic acid) to create an injectable hydrogel. Evaluation of the hydrogels showed favorable mechanical properties, rapid gelation, well injectability, and high cell viability and proliferation (Yan et al., 2016). In another study, a novel injectable hydrogel using PEG was synthesized for use in cartilage tissue engineering. The results showed adequate cartilage regeneration (Skaalure et al., 2015). However, one of the problems of synthetic biomaterials is lack of biological activity and low biocompatibility. To overcome these problems, synthetic biomaterials are modified or combined with bioactive biomaterials. For example, one study used a combination of hyaluronic acid and PEG to prepare an injectable hydrogel. The results showed that these hydrogels offer short gelation times, favorable mechanical properties, and high cell viability and proliferation. Therefore, it can be used for cartilage tissue engineering (Yu et al., 2014b).

### 2.2 Methods for manufacturing injectable hydrogel

Injectable hydrogels can be divided into three groups according to their manufacturing methods: chemical, physical, and microgels. Chemical hydrogels are formed by various methods, including Schiff base cross-linking, click chemistry, and enzymatic cross-linking. Physical hydrogels respond to various stimuli such as pH and temperature (Kim et al., 2009; Liu H. et al., 2016). Microgels (HMPs) can also be produced by various methods such as microfluidic emulsions, batch emulsions, EHD sputtering, mechanical fragmentation, and lithography (Sivashanmugam et al., 2015). In this section, we will look at three groups of injectable hydrogels and their respective manufacturing methods.

#### 2.2.1 Schiff-based cross-linked chemical hydrogels

**FIGURE 2:**
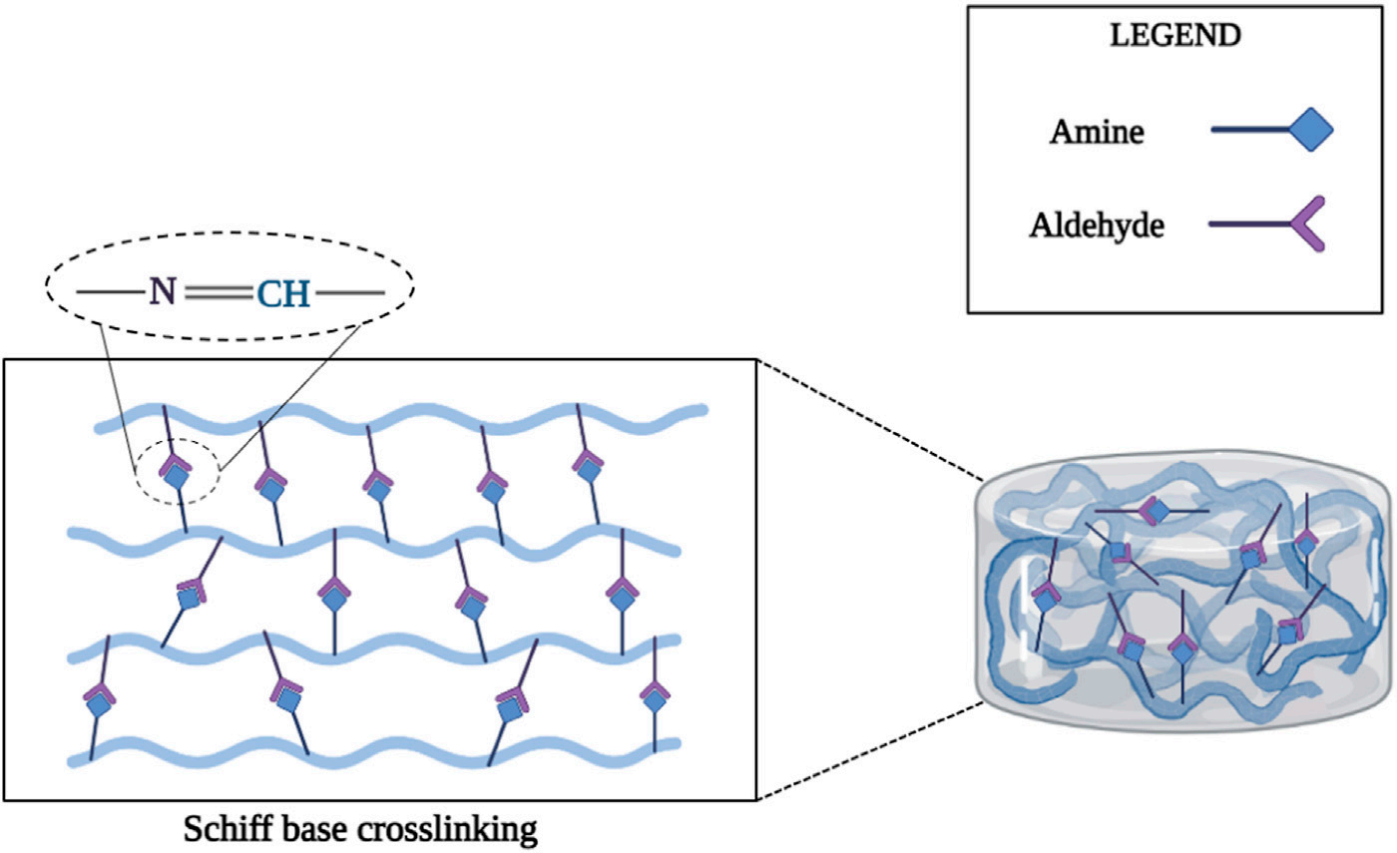
Schematic illustration of the production of injectable hydrogels by Schiff reaction.

The Schiff base reaction can form imine bonds between amino and aldehyde groups and has been studied for the synthesis of injectable hydrogels for cartilage tissue engineering applications due to its high reaction rate and mild reaction conditions (Zhang Y. et al., 2011; Xin and Yuan, 2012; Li Z. et al., 2015; Li L. et al., 2015; Ding et al., 2015). Chitosan is considered a desirable biological material for the fabrication of injectable Schiff base cross-linked hydrogels due to the presence of multiple amino groups in the main chain. For example, one study prepared an injectable chitosan-based hydrogel via the Schiff reaction between the aldehyde group of dextran and the amino group of chitosan for protein and cell delivery (Cheng et al., 2014). In another study, an injectable hydrogel was produced with a combination of poly (ethylene oxide-co-glycidol)-CHO and glycol chitosan through the Schiff reaction between the aldehyde groups of poly (ethylene oxide-co-glycidol)-CHO and the amino groups of glycol chitosan to repair damaged cartilage tissue (Figure 2) (Cao et al., 2015).

#### 2.2.2 Chemical hydrogels using click chemistry

Click chemistry includes several reactions such as tetrazine-norbornene chemistry (Alge et al., 2013), thiol-epoxy (Cengiz et al., 2013), thiol-ene reaction (Dong et al., 2012), and thiol-maleimide coupling (Arslan et al., 2014). Because of their low reactivity with cellular components and their rapid polymerization, these reactions have been studied to produce injectable hydrogels in cartilage tissue engineering (Hacker and Nawaz, 2015; Dong et al., 2016). For example, in one study, an injectable dendron-polymer-dendron conjugate hydrogel was prepared via a thiol-N-radical reaction. Sequential thiol–ene reactions use tetrathiol-based crosslinkers to crosslink polymer–dendron junctions to form transparent hydrogels (Kaga et al., 2016).

#### 2.2.3 Enzymatic cross-linking based chemical hydrogels

Recently, enzymatic cross-linking approaches have been considered as a new method for the production and development of injectable hydrogels due to their low cytotoxicity, stability under physiological conditions, and rapid gelation (Jin et al., 2014; Kuo et al., 2015). Various enzymes have been used to produce injectable hydrogels in cartilage tissue engineering, some of them are: Phosphatase, beta-lactamase, glutaminase, horseradish peroxidase (HRP), thermolysin, and tyrosinase (Teixeira et al., 2012). Among the mentioned enzymes, HRP is the most widely used enzyme for the production of injectable hydrogels. Horseradish peroxidase (HRP) is a β-type homologous protein that catalyzes working polymers (natural or synthetic) with aniline derivatives, phenolic compounds, and aminophenol molecules in the presence of H_2_O_2_ (Gohil et al., 2015; Hou et al., 2015). For example, in a study using HRP enzyme in the presence of H_2_O_2_, an injectable hydroxyphenylpropionic acid-gelatin hydrogel was synthesized for use in cartilage tissue engineering (Wang et al., 2014). Another study used Dex-TA in the presence of HRP and H_2_O_2_ to prepare an injectable hydrogel to repair damaged cartilage tissue (Jin et al., 2010b). Results showed survival and proliferation of type II collagen and glycosaminoglycans after culturing in the hydrogel for 21 days. Therefore, the injectable Dex-TA hydrogel cross-linked with HRP enzyme could be used for tissue engineering and damaged cartilage repair.

#### 2.2.4 Hydrogel microparticle or microgel

**FIGURE 3:**
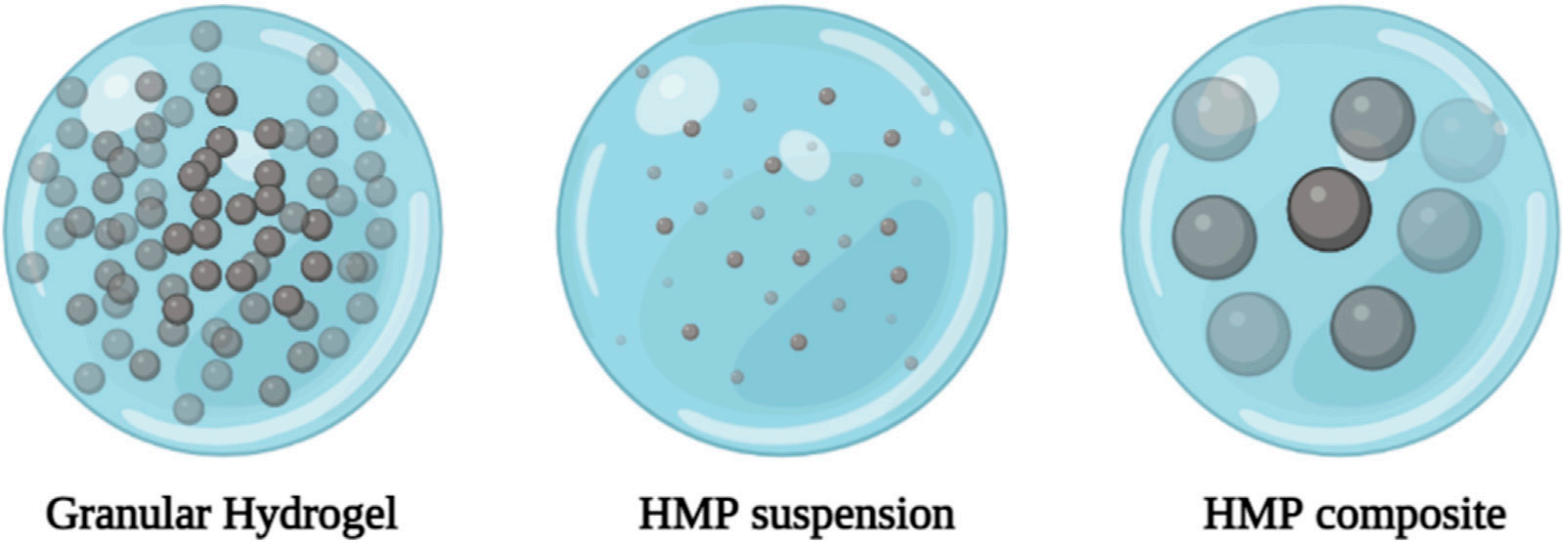
Schematic illustration of different types of hydrogel microparticles (HMPs).

HMP systems can generally be divided into three categories: granular hydrogels, HMP suspensions, and HMP composites (Figure 3) (Sivashanmugam et al., 2015). Various manufacturing techniques are used to manufacture HMPs, including: Microfluidic emulsions (Pittermannová et al., 2016; Headen et al., 2018; Zhang et al., 2018), batch emulsions (Franco et al., 2011; Leong et al., 2013), EHD deposition (Naqvi et al., 2016; Qayyum et al., 2017; Gansau et al., 2018), mechanical fragmentation (Sinclair et al., 2018), and lithograph (Pregibon et al., 2007; Le Goff et al., 2015) can be used. Typically, these methods involve forming droplets of hydrogel precursors and converting them into HMPs. Advanced technologies such as lithography, EHD deposition, and microfluidic emulsions provide better control of single particle formation and generate more monodisperse HMPs through changes in external and internal structures. However, methods such as mechanical grinding and batch emulsion are more widely used due to their faster production speed and simplicity. Additionally, mechanical grinding and batch emulsions require relatively simple equipment, whereas lithography, EHD deposition, and microfluidic emulsions require more advanced equipment. Key process parameters (particle size distribution, and particle formation rate) also vary depending on the production technology (Table 2) (Pregibon et al., 2007; Franco et al., 2011; Leong et al., 2013; Le Goff et al., 2015; Sivashanmugam et al., 2015; Naqvi et al., 2016; Pittermannová et al., 2016; Qayyum et al., 2017; Gansau et al., 2018; Headen et al., 2018; Sinclair et al., 2018; Zhang et al., 2018).

**TABLE 2:** Key parameters in different manufacturing techniques of HMPs.

Manufacturing techniques	Particle size distribution (μm)	Particle production rate	Cell compatibility during particle fabrication	Ref.
Microfluidic emulsion	5–10	Average	Average (>80% viability)	Pittermannová et al. (2016), Headen et al. (2018), Zhang et al. (2018)
Batch emulsion	1–10	High	Average (>80% viability)	Franco et al. (2011), Leong et al. (2013)
EHD sputtering	1–10	Average	Average (>80% viability)	Naqvi et al. (2016), Qayyum et al. (2017), Gansau et al. (2018)
Mechanical fragmentation	20–50	High	_	Sinclair et al. (2018)
Lithography	<1 possible	Low	High (>90%)	Pregibon et al. (2007), Le Goff et al. (2015)

## 3 Decellularized extracellular matrix-based hydrogels for cartilage tissue engineering

An ideal hydrogel for use in a biological application should exhibit properties that match the properties and structure of the extracellular matrix (ECM). Current polymer hydrogels show limitations in simulating ECM properties and functions (Zhang W. et al., 2021). In recent research in the field of repair and regeneration of biological tissues, decellularized ECM-based (dECM) hydrogels have been introduced as a promising approach. dECM hydrogels are a series of natural scaffolds based on organs and biological tissues whose cellular components have been removed while maintaining the 3D structure and some other components such as collagen fibers (Saldin et al., 2017; Giobbe et al., 2019). For prepare these hydrogels, first, ECM are turned into powder based on the freeze-drying method. Then, a certain ratio of the prepared powder is dissolved in an acidic solvent with a certain amount of acid protease to obtain a uniform solution. Next, by changing the pH and temperature of the solution or adding a crosslinking agent to it, hydrogel is formed (Freytes et al., 2008; Zhou et al., 2020). First time in 2008, Freytes et al. prepared a hydrogel based on decellularized ECM of pig bladder tissue (Freytes et al., 2008). After that, the use of dECM-based hydrogels in repairing and regenerating tissues such as kidney, heart, bone, cartilage, nerves, liver, and small intestine were studied (Saheli et al., 2018; Seo et al., 2018; Su et al., 2018).

These types of hydrogels have many advantages that make them suitable for pre-clinical and clinical applications. These advantages include: 1) mimicking the biological properties of the natural matrix. 2) injectability; dECM hydrogels can be injected into the biological environment using a syringe or catheter. 3) bioactivity. 4) They do not create immunogenicity. 5) Changing the plasticity according to the target tissue. 6) Their mechanical properties can be adjusted by changing the hydrogel concentration or by cross-linking. 7) These hydrogels can carry and support drugs, biologically active molecules (such as growth factors), and cells (Ungerleider et al., 2015; Lin et al., 2018).

Recently, the use of dECM-based hydrogels for cartilage tissue engineering applications has become a novel approach for the regeneration of damaged tissue. Due to the inherent properties of solubilized dECM, its combination with other biomaterials can produce hydrogels with tunable mechanical properties that induce chondrocyte growth and proliferation (Rothrauff et al., 2018). For example, in one study, a temperature-sensitive hydrogel based on decellularized porcine meniscal ECM was prepared for cartilage tissue engineering. *In vitro* results reported the growth of chondrocytes. In addition, the dECM solution was transformed into a hydrogel 30 min after subcutaneous injection into mice. The evaluation of the hydrogel confirmed the biocompatibility and lack of *in vivo* inflammatory reaction (Wu et al., 2015). In another study, articular cartilage-derived dECM was developed as an injectable vehicle for targeted drug delivery. *In vivo* results showed sustained release of bovine serum albumin over 10 days after subcutaneous injection in rats (Kwon et al., 2013b). According to these results, solubilized cartilage dECM can be introduced as a growing and promising system for carrying and delivering drugs, cells, and other bioactive molecules for cartilage tissue engineering applications.

## 4 Magnetic hydrogels for cartilage tissue engineering

Magnetic hydrogels (MHs) are a group of smart hydrogels that have recently been evaluated and used for tissue engineering and targeted drug delivery applications. These 3D structures are remotely controlled by an external magnetic field and can provide a platform for cell growth, proliferation, and migration (Taghizadeh et al., 2023). Drug delivery by MHs allows drugs to be transported directly to the target tissue due to the presence of a magnetic field. Also, the use of magnetic actuators in these systems creates remote control properties and controllable contraction of the hydrogel to release therapeutic agents (Ganguly and Margel, 2021). MHs are produced through the combination of magnetic agents and a hydrogel matrix. Nowadays, iron-based magnetic and biocompatible nanoparticles are used to combine with hydrogel matrix in various tissue engineering applications (e.g., bone, cartilage, heart, and nerves) (Pardo et al., 2021; Taghizadeh et al., 2023).

In cartilage tissue engineering applications, the magnetic properties of iron-based nanoparticles allow the magnetic control of injected cells in the damaged area through the mechanical manipulation of nanoparticles by an external magnetic field. In addition, *in vivo* studies have shown that bone marrow-derived stem cells (BMSCs) and chondrocytes labeled with magnetic iron nanoparticles can be easily detected by MRI systems non-invasively (Ramaswamy et al., 2009; Korchinski et al., 2015). However, factors such as manufacturing method, biocompatibility, and magnetic properties are influential in the selection of magnetic nanoparticles for the preparation of MHs. The most magnetic nanoparticles used in cartilage tissue engineering applications include; Maghemite (γ-Fe_2_O_3_) and magnetite (Fe_3_O_4_). Nevertheless, other nanomaterials such as neodymium iron boron (NdFeB), cobalt ferrite, and nickel ferrite have been introduced by researchers for cartilage tissue engineering applications (Taghizadeh et al., 2023). In the following, we review several published manuscripts on the application of MHs for cartilage tissue engineering as a new approach.

In one study, a theranostic hydrogel system based on grafting of kartogenin (KGN) with ultra-small superparamagnetic iron oxide (USPIO) nanoparticles was prepared to investigate the cartilage regeneration potential. KGN-USPIO nanocarrier loaded into a dextran/cellulose nanocrystal-based hydrogel to enhance cartilage regeneration. The results of *in vitro* and *in vivo* evaluation of this system showed that ideal mechanical properties and increased magnetic resonance contrast provide non-invasive monitoring of cartilage regeneration. In addition, *ex vivo* and *in vivo* studies demonstrated the long-term release of KGN from the hydrogel system, which induces the differentiation of MSCs into chondrocytes. These results demonstrate the potential of the KGN-USPIO hydrogel system for *in situ* cartilage regeneration (Yang et al., 2019). In another study, a hybrid hydrogel based on methacrylated gelatin (GelMA) chemically crosslinked with iron oxide nanoparticles (Fe_2_O_3_) with tunable stiffness properties was prepared. The results showed that GelMA/Fe_2_O_3_ hybrid hydrogel facilitates lipid catabolism in chondrocytes. Besides, it was found that GelMA/Fe_2_O_3_ hydrogel promotes mitochondrial oxidative phosphorylation. Moreover, *in vivo* studies in a mouse model with cartilage defects confirmed the potential of GelMA/Fe_2_O_3_ hybrid hydrogel for effective cartilage regeneration (Zhou et al., 2023). According to the results of the studies, magnetic hydrogels can be considered as a new and effective approach in cartilage tissue engineering applications.

## 5 Electroconductive hydrogels for cartilage tissue engineering

To date, the use of hydrogels based on different materials with various properties has been proven for cartilage tissue engineering applications. Among these, the use of conductive materials to make hydrogels has become a promising approach in tissue engineering. Even though the use of conductive materials has been investigated more in heart, skin, and nerve tissue engineering studies, nevertheless, in recent years, the use of these materials in cartilage tissue engineering has also attracted the attention of researchers (Gao et al., 2022; Miguel et al., 2022). These types of materials have appeared valuable for cartilage tissue engineering applications due to their common biophysical properties, including greater simulation of the physiological environment and electrical stimulation (Lee et al., 2022). Recently, based on the electrical properties identified from articular cartilage and the positive results obtained from the effect of electrical stimulation of the tissue to increase the differentiation of mesenchymal stem cells into chondrocytes and repair and regeneration of cartilage tissue, many research studies have been conducted in this field (Vaca-González et al., 2016; Hernández-Bule et al., 2017; Vaca-González et al., 2020).

The underlying mechanisms by which endogenous or external electrical stimulation affects cell behavior are still not understood. However, electrical stimulation changes the resting potential of the cell membrane, causing voltage-gated calcium channels (VGCC) to open, allowing cells to take up calcium, which is one of the main responses. Increased calcium in cells activates calcineurin and calmodulin-mediated signaling pathways, thereby altering the gene expression profile of the cell and inducing the production of chondrogenesis-associated growth factors such as TGF-β and BMPs (Thrivikraman et al., 2018). Combining electrical stimulation with TGF-β1 and BMP-2 inhibitors, Kwon et al., 2016 found that the concentration of MSCs required for chondrogenesis was better after electrical stimulation, a process mediated by TGF-β signaling. Additionally, activation of the mitogen-activated protein kinase (MAPK) signaling pathway is another way that electrical stimulation modulates cell behavior (Sun et al., 2015). However, although further studies are needed to understand the mechanism of electrical stimulation-mediated AC generation, using conductive hydrogels to create tissue microenvironment *in vitro* is promising, and improving the current system is also a good idea.

In one study, polyvinyl alcohol (PVA)-based hydrogel combined with sodium phytate (PANa) was synthesized for articular cartilage repair. Researchers confirmed that the presence of PANA in the hydrogel gave it excellent mechanical and conductive properties. The tensile strength of the hydrogel reported through the rheology test was higher than 7 MPa, which could show a resistance of more than 600% against the strain. The reported conductivity properties for PVA/PANa hydrogel is about 1.65 S/m, which according to experiments corresponds to the conductivity of articular cartilage (approximately 1.2 S/m). Finally, PVA/PANa hydrogel can be used as a suitable conductive system in the application of articular cartilage regeneration (Zhang S. et al., 2021). In another study, Distler et al., 2021 prepared a polypyrrole/polystyrene sulfonate (PPy/PSS) modified oxidized alginate-gelatin (ADA-GEL) based hydrogel for cartilage tissue engineering using 3D bioprinting technique. The results of the evaluation of mechanical properties showed that hydrogels with a concentration of 0.1 M PPy showed a tensile strength of approximately 1.2 MPa at a strain of 40%, which was the best among other hydrogels. In addition, the conductivity properties of 0.1 M PPy hydrogel were reported between 1 and 1.4 S/m, which is similar to the properties of local cartilage. Also, researchers were able to increase the efficiency of cell culture in hydrogel by using 3D bioprinting technique. According to these results, the hydrogel made by Distler et al. can provide a promising approach in the field of articular cartilage repair.

## 6 Hydrogel-guided delivery for gene vectors for targeted cartilage repair

Controlled gene delivery through biomaterials to repair damaged cartilage prevents vector degradation and enhances the temporal and spatial effects of the genetic product. However, the use of controlled gene delivery methods is still considered a major challenge (Cucchiarini, 2016). Combinatorial gene delivery approaches using hydrogels, scaffolds, and micelles have been explored (Cucchiarini and Madry, 2019). For example, in a study, a fibrin-based hydrogel carrying the rAAVTGFB1 gene was prepared to repair and repair damaged cartilage tissue. The results showed increased expression of cartilage tissue-specific genes in mesenchymal stem cells (ACAN and SOX9) (Lee et al., 2011). In another study, a poloxamine-poloxamer hydrogel was developed to deliver rAAV-TGFB1 vector. Results showed increased deposition of type II collagen and proteoglycans (Rey-Rico et al., 2017). In another study, poly (ethylene oxide)-b-poly-L-lysine and/or PLGA-fibrin gels were used to fabricate hybrid scaffolds for nonviral delivery of TGFB1. Both biomaterials used showed integration with surrounding cartilage tissue and restoration of damaged cartilage (Li et al., 2013; Li et al., 2014). As a result, a combined approach of gene delivery using hydrogels and hydrogel scaffolds may be useful for cartilage tissue repair and regeneration, but these approaches require further research.

## 7 Biomimetic fibrillar hydrogels for cartilage tissue engineering

ECM-mimicking hydrogels are used for 3D cell culture and tissue engineering of cartilage, bone, and skin. by providing suitable biochemical and biophysical properties. Fibrillar hydrogels have a fibrous structure that mimics the shape and fibrillar pattern of extracellular matrix (ECM). The fibrous structure of these types of hydrogels influences pore size, migration, and mechanical properties (Prince and Kumacheva, 2019). Biomimetic fibrillar hydrogels are formed in a one-step process by combining block copolymers (Blanazs et al., 2012; Simon et al., 2015; Warren et al., 2015), cellulose nanofibers (De France et al., 2017; Li et al., 2017), peptides (Sur et al., 2013; Morgan et al., 2016), chitosan and cellulose (Liu M. et al., 2016; Li et al., 2017) as hydrogel building blocks.

In this section, we review some biomimicking fibrillar hydrogels made from natural and synthetic biomaterials.

### 7.1 Peptide fibrillar hydrogels

Peptides can form fibrils through various types of non-covalent interactions such as hydrophobic, hydrogen, van der Waals interactions, and electrostatic forces, and when the concentration and length of fibrils are sufficiently increased, they form fibril-type hydrogels (Thérien-Aubin et al., 2016). Peptide-based fibrillar hydrogels can be divided into two categories. 1) peptide-based fibrillar hydrogels with native structures (forming alpha helix and beta sheets) and 2) peptide-based fibrillar hydrogels modified to promote self-assembly (with non-basic abilities) (Prince and Kumacheva, 2019). The main advantage of fibrillar peptide hydrogels is their modularity, which allows their properties to be tuned by changing their amino acid sequences (Pashuck et al., 2010; Stupp, 2010).

### 7.2 Fibrillar hydrogels based on cellulose nanofibers

Cellulose nanofibers are available primarily as bundles of narrow fibrils with diameters of 5–40 nm and approximately several micrometers in length. These biomaterials have a semicrystalline structure (amorphous and crystalline structures), and the degree of crystallinity varies between 65% and 95% depending on conditions (Saito et al., 2007; Sehaqui et al., 2011). Unlike peptide-based fibrillar hydrogels, cellulose nanofiber-based fibrillar hydrogels are formed by bonding and entanglement. Monocellulose nanofibers are formed by TEMPO (2,2,6,6-tetramethylpiperidine-1-oxyl) oxidation reaction (Saxena and Brown, 2005; Prince and Kumacheva, 2019). Fibrillar hydrogels based on cellulose nanofibers can be used as 3D scaffolds in engineering areas such as cartilage tissue, liver (for keloid growth from liver cells), and skin (Lou et al., 2014).

### 7.3 Fibrillar hydrogels based on cellulose and chitin

Nanocrystals of cellulose and chitin are nanoparticles with a rod-shaped structure with a diameter of 5–30 nm and a length of 100–300 nm (Liu M. et al., 2016). Unlike cellulose nanofibers, these nanocrystals are not long enough and cannot form networks through entanglement. However, nanocrystals can be attached end-to-end to fibers of different diameters (Chau et al., 2015). Cellulose nanocrystals are formed by acid hydrolysis of cellulose nanofibers and have high mechanical properties (flexural strength of approximately 10 GPa, Young’s modulus of 100–130 GPa) (Abe and Yano, 2011; Sanna et al., 2013). An effective and cytocompatible strategy for the formation of fibrillar hydrogels is to combine a gelling polymer with cellulose-nanocrystal-derived hydrogels. For example, poly (N-isopropyl acrylamide-co-2-(dimethyl amino)ethyl methacrylate) and poly (N-isopropyl acrylamide) (pNIPAM) grafted cellulose nanocrystals transform into fibers that They produce gel at a polymer low critical temp (Schneider et al., 2002; Li et al., 2017). These hydrogels can also be studied and used in cartilage tissue engineering, skin fibroblast culture, and the growth of breast cancer spheroids (Li et al., 2017).

According to the information mentioned in this section, biomimetic fibrillar hydrogel can be used as an effective biomaterial for cartilage tissue engineering.

## 8 Stimuli-responsive hydrogels for cartilage tissue engineering

Hydrogels can be considered smart biomaterials due to their sensitivity to various stimuli (e.g., pH and temperature) (Imran et al., 2010). This part of the study explores injectable stimuli-responsive hydrogels and their applications in cartilage tissue engineering.

### 8.1 PH-responsive injectable hydrogels

Preparation of injectable pH-responsive hydrogels requires pH-sensitive moieties such as polyacrylic acid (PAA) or sulfamethazine oligomer (SMO) (Huynh et al., 2012; de Lima et al., 2015). For example, in a study, a pH-sensitive injectable hydrogel was prepared by combining (PCLA-PEG-PCLA) and adding pH-sensitive SMOs to both ends of the polymer composition (SMO-PCLA-PEG-PCLA-SMO) (Kim et al., 2009). Results showed the stability of the gel and differentiation of mesenchymal stem cells (7 weeks after transplantation) under physiological conditions (pH = 7.4).

### 8.2 Temperature-responsive injectable hydrogels

Recently, thermoresponsive injectable hydrogels have attracted widespread interest in cartilage tissue engineering due to their ability to form gels at physiological temperatures. These injectable hydrogels are liquid at room temperature but quickly change to a gel at ambient physiological temperatures (Sood et al., 2016). Temperature-sensitive hydrogels can change phase without chemical stimulation, which is their most valuable feature. The phase transition process in injectable hydrogels responds to temperature in a way that changes the uptake (hydration) of available water when the temperature changes, causing changes in the solubility of the hydrogel (Yu and Zheng, 2011; Ashraf et al., 2016). To prepare injectable temperature-responsive hydrogels, polymers such as poly (N, N-diethylacrylamide) (Sood et al., 2016), and PNIPAAm (Ashraf et al., 2016), which respond to temperature stimuli, are used. PNIPAAm is one of the most common temperature-sensitive polymers. This polymer is derived from polyacrylic acid and exhibits a rapid phase transition at temperatures below 32°C (Duarte Campos et al., 2012; Hu et al., 2015b; Vo et al., 2015). However, this polymer is not stable at body temperature. A strategy to solve this problem and improve mechanical properties is to modify the polymer with other natural polymers. For example, in an atom transfer radical polymerization study, gelatin was grafted onto PNIPAAm and an injectable hydrogel was fabricated that underwent a sol–gel transition at physiological temperature (Ren Z. et al., 2015). The results showed that the hydrogel was biocompatible and non-toxic. Therefore, this hydrogel may be suitable for cartilage tissue engineering applications.

## 9 Cartilage-targeting drug delivery

Electrostatic interactions (charge-charge) can be explored as a new approach for targeted drug delivery to negatively charged tissues (such as cartilage) and the treatment of early-stage osteoarthritis (which is a reversible and treatable disease) (Bajpayee and Grodzinsky, 2017). In this method, cationic nanocarriers (positive charge) with sizes of 10 nm or less are produced, which can penetrate the cartilage tissue (Bajpayee et al., 2014). The positive charge in these nanocarriers leads to an increase in the transport speed and shortens the treatment time in the cartilage tissue (Hu H.-Y. et al., 2015). For example, in one study, dexamethasone was combined with cationic avidin carriers. Avidin penetrated the cartilage tissue due to its load properties and favorable size and released the drug (dexamethasone) inside the tissue (Gouze et al., 2004). In another study, cationic nanocarriers based on DOTAM (1,4,7,10-tetra aza cyclo dodecane-1,4,7,10-tetra acetic acid amide) were functionalized with cathepsin D. The results of *in vivo* studies showed the durability of the drug in the knee joint of mice (Coombe, 2008).

Other approaches to treating damaged cartilage and targeting chondrocytes have been explored. In these approaches, fibroblast growth factor (FGF) and vascular endothelial growth factor (VEGF) proteins have been used to bind cations to heparin and heparan sulfate (Loffredo et al., 2014). For example, in one study intra-articular injection of HB-IGF-1 resulted in increased local migration to cartilage cells and greater biological activity in cartilage tissue (Miller et al., 2010; Grodzinsky et al., 2017). Therefore, cationic drugs also use electrostatic interactions to transport growth factors into the cartilage tissue at a faster rate (Bajpayee and Grodzinsky, 2017). Finally, electrostatic interactions can be exploited for targeted drug delivery to cartilage tissue for the treatment of OA.

## 10 Mechanosignaling in cartilage treatment

**FIGURE 4:**
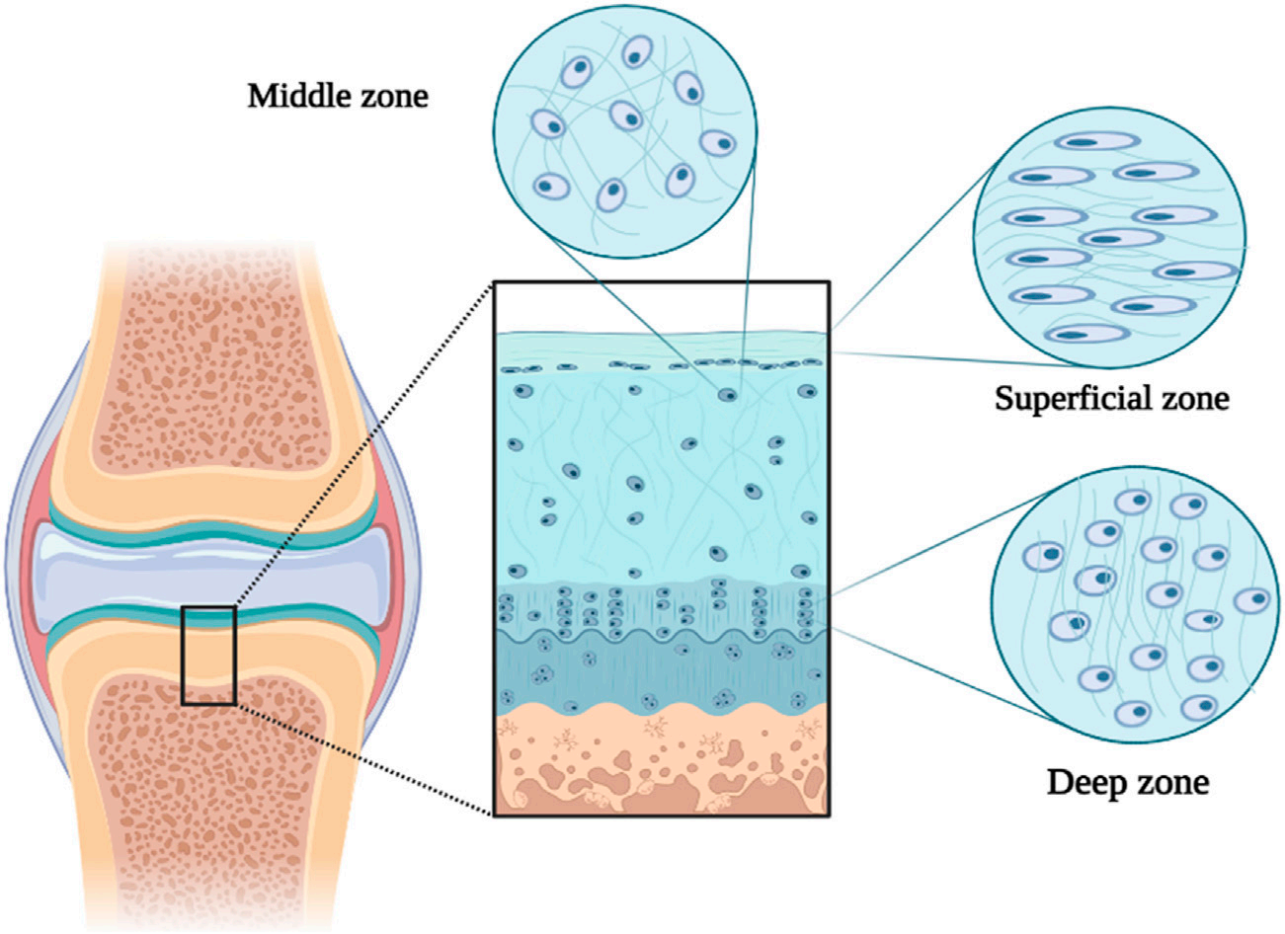
Schematic illustration of different parts of articular cartilage.

Mechanical signaling is considered an important and effective pathophysiological and physiological process in cells and cartilage tissue. Movement is important for maintaining healthy cartilage and preventing OA (Hodgkinson et al., 2022; Pisheh et al., 2022). Articular cartilage (from the subchondral bone to the joint surface) is divided into three deep, middle, and superficial parts (Poole et al., 2001). The structure and composition of the cartilage ECM cause the tissue to bear various loads (such as shear, compression, and transverse) during movement. For example, In the surface part of the tissue, type II collagen fibers and cartilage cells (chondrocytes) are placed transversely to divide and disperse shear forces. Since the forces enter compressive and shearing forms in the middle part of the tissue, type II collagen fibers are placed randomly and scattered. In the deep part of the tissue, the vertical arrangement of thick type II collagen fibers and the increase in the concentration of proteoglycan lead to bearing compressive loads in movement (Figure 4) (Poole et al., 2001; Pap and Korb-Pap, 2015). Therefore, cartilage ECM destruction can also affect the cellular response due to the effect of the forces applied to the tissue (Burleigh et al., 2012). Around chondrocytes, there is a softer area of ECM with a thickness of 2–4 μm called the pericellular matrix (PCM), which has the greatest effect on the mechanical transmission of cells and directly affects the balance of mechanical forces applied to the cell. Therefore, the destruction of PCM can be effective in the development of OA (Poole, 1997; Guilak et al., 2018). Cartilaginous cells respond to direct and indirect mechanical stresses by sensing deformation in PCM or mechanical release of growth factors.

This section examines direct and indirect mechanical signaling mechanisms such as signaling through integrins and growth factors (Loeser, 2002; Millward-Sadler and Salter, 2004; Ross et al., 2013).

### 10.1 Mechanosignaling by integrins

Among the cell adhesion molecules, integrins have a significant effect in determining the cellular response to the surrounding environment. Integrins are heterodimers. Different types of α and β integrins (such as α1β1, α5β1, α10β1, and α11β1) exist in articular cartilage (Salter et al., 1992; Zhang et al., 2002; Gouttenoire et al., 2010). Expression of some integrin subunits causes mechanical signaling in healthy cartilage. For example, in an *in vivo* study, αV integrin deletion in mouse chondrocytes showed reduced ECM degradation as well as reduced transforming growth factor beta (TGFβ) activation. Under the mechanical stress present in OA, integrin αV causes tissue ECM degradation by activating TGFβ signaling. When the mechanical stress increases in the tissue, the αV integrin is activated, and this activation causes contraction forces to be applied on the matrix by cartilage cells and increase cell stiffness. As a result, stiffness in chondrocytes leads to the activation of TGFβ by αV in the tissue (Zhen et al., 2021). In addition, TGFβ increases the expression of integrin α11 subunits in cartilage, and the expression of integrin α11 also leads to the differentiation of mesenchymal stem cells (MSCs) (Varas et al., 2007; Gouttenoire et al., 2010). For example, in one study MSCs were used to overexpress integrin α10β1. The results led to the reduction of cartilage tissue fibrillation and the production of subcartilaginous bone sclerosis (Delco et al., 2020). A new and attractive strategy to activate integrins is the re-use of integrin receptor antagonists in the treatment of OA. For example, it has been shown that a selective inhibitor of αVβ5 and αVβ3 integrins, cilengitide, can be used to treat glioblastoma by suppressing inflammatory mediators such as IL-1β and TNF in mouse ATDC5 chondrocytes (Hirose et al., 2020).

### 10.2 Mechanosignaling by growth factors

By changing the structure or destruction of PCM, growth factors such as FGFs, TGFβ, and bone morphogenic protein (BMP) are activated and communicate with cell membrane receptors, thus activating intracellular signaling (Martin et al., 2002; Guilak et al., 2006; Youn et al., 2006). The signaling of FGFs by PCM can be considered a suitable example of mechanical signaling by growth factors. All FGFs depend on heparin sulfate as a common receptor suitable for binding and activating FGF receptors (FGFRs) (Vincent, 2011; Xie et al., 2020). The effects of signaling by FGFs depend on FGF receptors and members of the FGF family. For example, recombinant human FGF18 (named sprifermin) by activating the FGFR3 receptor leads to an increase in cartilage thickness after intra-articular injection in patients with OA (Lohmander et al., 2014; Eckstein et al., 2015; Hochberg et al., 2019). Of course, the topic of signaling by growth factors (such as FGFs) needs more investigation, but according to the said material, this strategy can be mentioned as a method for developing OA drug treatment.

### 10.3 Engineered hydrogels for cartilage mechanobiology

Hydrogels have already proven invaluable in developing a fundamental understanding of how internal mechanical cues influence fate decisions. For example, altering 2D surface stiffness can direct human mesenchymal stem cell (hMSC) differentiation in the absence of differential soluble signals (Walters and Gentleman, 2015; Blache et al., 2022). Differentiation was most effective on surfaces that matched the stiffness of the underlying tissue. This suggests that to create regenerative therapies, the biomaterial scaffold must perfectly match the stiffness of the underlying tissue it is intended to replace. Similarly, stem cells isolated from skeletal muscle and grown on matrices matching the stiffness of the underlying tissue self-renew *in vitro* and promote muscle regeneration *in vivo* (Engler et al., 2006). This contrasts with the regenerative capacity during plastic tissue culture, where progenitor cells lose their ability to reproduce and repair. Therefore, hydrogels that can provide mechanical signals to cells have been used to understand fundamental biological processes and control cell behavior for regenerative purposes, including the repair of cartilage, bone, and muscle (Kim S. et al., 2021). For example, chondrocytes encased in a rapidly relaxing ionic cross-linked hydrogel expand in volume and secrete extensive cartilage-like matrix. Conversely, confined hydrogels that do not allow cell expansion allow encapsulated chondrocytes to activate genes associated with cartilage catabolism (Lee et al., 2017).

## 11 Challenges and future perspectives

Today, tissue engineering (here cartilage tissue engineering), is one of the conventional, new, and minimally invasive methods for treating many different diseases such as bone and joint diseases (cartilage tissue), cardiovascular diseases, skin diseases, and kidney diseases. Due to the spread and prevalence of tissue engineering in today’s world, biomaterials and therapeutic methods for the application of cartilage tissue repair and regeneration are also increasing. According to the surveys conducted in the past few years, fabricated injectable hydrogels for the repair and regeneration of cartilage tissue have received much attention. However, the production and evaluation of these injectable hydrogels face different challenges, including the type of biomaterials and manufacturing methods. The most important challenge in fabricating these hydrogels is to design hydrogels with tissue-compatible mechanical properties, biological stability, appropriate biodegradability, and high biocompatibility that show the ability to culture various cells. Also, they should be able to be used as suitable carriers for drugs and growth factors delivery. To overcome these challenges and propose the best option, we first investigate some biomaterials (natural and synthetic) used in fabricating injectable hydrogels such as collagen/gelatin, alginate, chitosan, heparin, chondroitin sulfate, polyvinyl alcohol (PVA), PEG, polypropylene fumarate (PPF) and other cases. Injectable hydrogels based on natural biomaterials, despite their advantages such as low cytotoxicity, high biocompatibility, and imitating the structure of natural tissue (cartilage), lack the desired mechanical strength limits their use. On the contrary, injectable hydrogels based on synthetic biomaterials show good mechanical properties but have lower bioactivity and biocompatibility. After that, we examined the methods of fabricating injectable hydrogel systems (physical, and chemical). Injectable hydrogels that are made by chemical methods, despite their high mechanical properties and stability, suffer from disadvantages such as the adverse effects of chemical reactions. Also, injectable hydrogels fabricate by physical methods face advantages such as sensitivity to various stimuli (PH and temperature), low cytotoxicity, and easy production, with disadvantages such as low stability and short-term response. Therefore, biomaterials (both natural and synthetic) and the methods mentioned in this study, to achieve new and desirable injectable hydrogels in cartilage tissue engineering, still need more investigation for clinical applications, drug delivery approaches based on injectable hydrogels and developing and expanding mechanobiology methods used in cartilage tissue engineering.
